# Calretinin is a novel candidate marker for adverse ovarian effects of early life exposure to mixtures of endocrine disruptors in the rat

**DOI:** 10.1007/s00204-020-02697-3

**Published:** 2020-03-27

**Authors:** Hanna Katarina Lilith Johansson, Terje Svingen, Julie Boberg, Paul A. Fowler, David Stead, Anne Marie Vinggaard, Panagiotis Filis

**Affiliations:** 1 Division of Diet, Disease Prevention and Toxicology, National Food Institute, Technical University of Denmark, 2800 Kongens Lyngby, Denmark; 2 Institute of Medical Sciences, School of Medicine, Medical Sciences and Nutrition, University of Aberdeen, Foresterhill, Aberdeen, AB25 2ZD UK

**Keywords:** Ovary, Proteome, Endocrine disruption, Calretinin, Ovarian dysgenesis syndrome, AOP

## Abstract

Disruption of sensitive stages of ovary development during fetal and perinatal life can have severe and life-long consequences for a woman’s reproductive life. Exposure to endocrine disrupting chemicals may affect ovarian development, leading to subsequent reproductive disorders. Here, we investigated the effect of early life exposure to defined mixtures of human-relevant endocrine disrupting chemicals on the rat ovary. We aimed to identify molecular events involved in pathogenesis of ovarian dysgenesis syndrome that have potential for future adverse outcome pathway development. We therefore focused on the ovarian proteome. Rats were exposed to a mixture of phthalates, pesticides, UV-filters, bisphenol A, butyl-paraben, and paracetamol during gestation and lactation. The chemicals were tested together or in subgroups of chemicals with anti-androgenic or estrogenic potentials at doses 450-times human exposure. Paracetamol was tested separately, at a dose of 360 mg/kg. Using shotgun proteomics on ovaries from pup day 17 offspring, we observed exposure effects on the proteomes. Nine proteins were affected in more than one exposure group and of these, we conclude that calretinin is a potential key event biomarker of early endocrine disruption in the ovary.

**Electronic supplementary material:**

The online version of this article (10.1007/s00204-020-02697-3) contains supplementary material, which is available to authorized users.

## Introduction

The foundation for a woman’s reproductive health is set during fetal life and hinges on the development of germ cells and functional ovaries. The ovaries are the primary female sex organs responsible for the production of eggs and sex hormones. They are central for reproductive health throughout adulthood, but are also involved in fetal and perinatal development of the female reproductive tracts and female sex characteristics. Disruption of ovarian development or function during early life stages can, therefore, have severe consequences later in life, affecting both fertility and health more broadly (Palmer et al. 2001; Hatch et al. 2006; Steiner et al. 2010; Hoover et al. 2011; Faubion et al. 2015).

The ovarian dysgenesis syndrome hypothesis states that many late-life reproductive disease manifestations arise from disrupted ovary development (Crain et al. 2008; Buck Louis et al. 2011; Johansson et al. 2017). Central to the ovarian dysgenesis syndrome hypothesis is that exposure to environmental chemicals during fetal life is a contributing factor to late-life disease. A growing body of evidence indicates that intrauterine exposure to selected chemicals perturbs ovarian development and function, and possibly affects the reproductive system later in life (Buck Louis et al. 2011; Johansson et al. 2017). There are, however, many challenges when linking fetal exposure to reproductive disorders in adult life. First, there is a significant lag time between exposure and disease manifestation, not least in humans. Second, the ovarian dysgenesis syndrome hypothesis comprise a complex group of phenotypes that also vary greatly in time of onset; that is, they represent a complex disease pattern. Third, humans are exposed to a complex, and constantly changing, mixture of chemicals throughout life.

Identification of the mechanisms behind later life effects after fetal exposure is a challenging task. Linking fetal exposure to reproductive disorders in adult life require that we know which mechanisms were affected in the fetus during exposure. Omics methods have proved invaluable for identifying new mechanisms and regulatory pathways within different fields (Frost and Amos 2018; Liu et al. 2019; Myburg et al. 2019). Omics approaches have also been taken up by the field of toxicology, as they offer new ways of integrating molecular events with health risk assessment (Brockmeier et al. 2017; Darde et al. 2018; Filis et al. 2019). Omics approaches are particularly attractive for developing novel adverse outcome pathways (AOPs), which are conceptual frameworks that describe causative links between molecular events and adverse outcomes at the biological level of organization (Ankley et al. 2010). The combination of omics and the AOP conceptual framework presents an important approach to focus on the key molecular events in female reproductive disease etiology.

We have performed a proteomic analysis of rat ovaries collected from offspring exposed to complex mixtures of endocrine disrupting chemicals via their mother during fetal and postnatal life. These offspring displayed ovarian dysgenesis syndrome-like symptoms when they had reached adulthood, with adverse outcomes including altered follicle numbers, abnormal mammary gland outgrowth, as well as advanced pubertal onset (Mandrup et al. 2015; Johansson et al. 2016). In aging animals, estrous cycling, number of corpora lutea, and mammary gland histology were adversely affected (Isling et al. 2014; Mandrup et al. 2015; Johansson et al. 2016). Thus, our aim was to identify putative molecular events that are involved in the ovarian dysgenesis syndrome pathogenesis and exhibit potential for future AOP development.

## Materials and methods

### Chemicals

Chemicals were: di-*n*-butyl phthalate (DBP) (purity > 99.0%, CAS no. 84-74-2), di-(2-ethylhexyl) phthalate (DEHP) (purity > 99.5%, CAS no. 117-81-7), vinclozolin (purity > 99.5%, CAS no. 50471-44-8), prochloraz (purity > 98.5%, CAS no. 67747-09-5), procymidone (purity > 99.5%, CAS no. 32809-16-8), linuron (purity > 99.0%, CAS no. 330-55-2), epoxiconazole (purity > 99.0%, CAS no. 106325-08-8), octyl methoxycinnamate (OMC) (purity > 98.0%, CAS no. 5466-77-3), dichlorodiphenyl-dichloroethylene (p,pʹ-DDE) (purity > 98.5%, CAS no. 72-55-9); all purchased from VWR-Bie & Berntsen (Herlev, Denmark). 4-methyl-benzylidene camphor (4-MBC) (purity > 98.0%, CAS no. 36861-47-9), bisphenol A (BPA) (purity > 99.5%, CAS no. 80-05-7), butyl paraben (purity > 99.0%, CAS no. 94-26-8) and paracetamol (PM) (purity > 99.0%, CAS no. 103-90-2) were all purchased from Sigma-Aldrich (Brøndby, Denmark). Corn oil was used as a control compound and as vehicle; purchased from VWR-Bie & Berntsen (Herlev, Denmark).

### Chemical mixtures

The composition of the mixtures was based on high-end human exposure levels, with the mixture designed as previously described (Christiansen et al. 2012; Axelstad et al. 2014). In short, the Totalmix contained all 13 compounds, the AAmix contained compounds considered to have predominantly anti-androgenic modes of action, and the Emix contained compounds considered to have predominantly estrogenic properties (Table 1). PM was included in the Totalmix, as well as tested on its own, but was not included in the AAmix nor the Emix. The mixtures were administered at 450-times human high-end exposure. These doses were predicted to affect anti-androgenic endpoints in male offspring (see Christiansen et al. 2012). Doses were higher than human exposure levels to increase the chance of registering a response from subtle effects that would otherwise not be detected, using a relatively small number of animals in a rat model with more rapid metabolism of chemicals than humans. PM was administered at 360 mg/kg, both in the Totalmix and in the single exposure, corresponding to human exposure levels when taking into account toxicokinetic differences between rats and humans (Table 1).

**Table 1:** Mixture composition and dose for the tested mixtures in mg/kg per day. Design of the mixtures has previously been described (Axelstad et al. 2014; Christiansen et al. 2012)

Chemical	Mixture dose (mg/kg per day)			
	Totalmix 450	AAmix 450	Emix 450	PM
DBP	4.5	4.5	0	0
DEHP	9	9	0	0
Vinclozolin	4.05	4.05	0	0
Prochloraz	6.3	6.3	0	0
Procymidone	6.75	6.75	0	0
Linuron	0.27	0.27	0	0
Epoxiconazole	4.5	4.5	0	0
*p,p*ʹ-DDE	0.45	0.45	0	0
4-MBC	27	0	27	0
OMC	54	0	54	0
Bisphenol A	0.675	0	0.675	0
Butyl paraben	27	0	27	0
Paracetamol	360	0	0	360

### Animals and exposure

The animal study is described in (Axelstad et al. 2014). In short, time-mated nulliparous Wistar rats (HanTac:WH, SPF, Taconic Europe, Ejby, Denmark) were used and the day of vaginal plug detection designated GD 1, and the expected day of delivery (GD23) designated pup day (PD) 1. The dams were supplied at GD 3. Animals were exposed to vehicle (controls), or one of the four mixtures (Table 1). Each dose group comprised 16–20 dams, and 14–20 viable litters were obtained for each group. Rats were exposed by oral gavage from GD7-21, and again after birth from PD1-22. Exposure to PM was from GD13-19 and PD14-22, both in the Totalmix and single dosing, meaning that only PM was given in a different time interval. This was to avoid effects on embryo implantation (Gupta et al. 1981) and problems during parturition. The study was performed under conditions approved by the Danish Animal Experiments Inspectorate (Council for Animal Experimentation) and by the in-house Animal Welfare Committee.

### Protein extraction and processing

Ovaries were collected from PD17 female offspring as these animals are in a period of rapid development and all stages of developing follicles (primordial to antral follicles) are present. Also, in comparison to later ages, offspring is still fully lactating and thus receive chemicals through maternal milk. Ovaries were snap-frozen in liquid nitrogen, then stored at − 80 °C until processing. Proteins were isolated from 8 to 9 individual ovaries per group (different litters) using the AllPrep kit (#80004; QIAGEN, Manchester, UK) according to the manufacturer’s instructions. Ovaries were extracted individually and all further analyses were conducted on individual samples. Protein concentrations were quantified using a modified Lowry assay (Biorad Ltd., Hertfordshire, UK, cat.no. 500–0122). 10 μg of protein extracts were diluted to a final volume of 100 μl of 50 mM NH_4_HCO_3_ (BioUltra grade, Sigma Aldrich). Proteins were digested in solution according to the PRIME-XS protocol. Briefly, proteins were reduced in 2 mM dithiothreitol (Sigma Aldrich, > 99%) for 25 min at 60 °C and *S*-alkylated in 4 mM iodoacetamide (Sigma Aldrich, > 99%) for 30 min at 25 °C in the dark, then digested by sequencing-grade modified trypsin (Promega, Southampton, UK, cat.no. V5111) at a 1:10 ratio of trypsin:protein overnight at 37 °C. The reaction was stopped by freezing at − 80 °C. Samples were then thawed, dried by vacuum centrifugation (SpeedVac Plus SC110A, Savant) and dissolved in 10 µL 2% acetonitrile/0.1% formic acid. The equivalent of 2 μg of peptides (assuming no losses) were analysed by liquid chromatography-tandem mass spectrometry (LC–MS/MS). The LC–MS system comprised a Thermo Scientific Dionex UltiMate 3000 RSLC nano-LC configured for pre-concentration onto a nano column, coupled to a Q Exactive Plus hybrid quadrupole-Orbitrap mass spectrometer fitted with an EASY-Spray nano-ESI source (Thermo Scientific). Peptide samples were injected onto a C18 PepMap 100 pre-column (300 µm i.d. × 5 mm) in loading pump solvent (2% acetonitrile, 0.1% formic acid) at a flow rate of 10 µL/min for 5 min. The pre-column was then reverse-flushed to the analytical column (PepMap RSLC C18; 50 µm i.d. × 15 cm; Nano pump solvent A: 0.1% formic acid, Nano pump solvent B: 80% acetonitrile, 0.1% formic acid) at 0.3 µL/min using the nano pump. Peptides were separated using a gradient of acetonitrile (LC gradient: 3–10% solvent B in 5 min, 10–40% solvent B in 30 min, 40–80% solvent B in 5 min, hold at 80% solvent B for 8 min, 80–3% solvent B in 1 min, hold at 3% solvent B for 15 min) while MS/MS data were acquired by the Q Exactive in data-dependent mode (Top10 method). Parameters for the full scan/data-dependent MS2 (Top10) method were: full scan range 375–1750 *m*/*z*; resolution 70,000; AGC target 3e6; maximum IT 50 ms. MS2 scan resolution 17,500; AGC target 5e4; maximum IT 100 ms; loop count 10; isolation window 1.6 *m*/*z*; NCE 26; underfill ratio 4%; charge states 2–5 included; peptide match preferred; exclude isotopes on; dynamic exclusion 40 s. Technical replicates for 25% of the samples were performed. The mass spectrometry proteomics data have been deposited to the ProteomeXchange Consortium via the PRIDE (Perez-Riverol et al. 2019) partner repository with the dataset identifier PXD015470.

### Analysis of LC–MS/MS output

RAW Q-Exactive output files were processed in 4 runs (controls vs each of the four exposures) by MaxQuant (v 1.5.3.30) (Cox and Mann 2008). MaxQuant runs were performed under the default parameters except (a) trypsin was set as the digestion enzyme; (b) asparagine (N) deamidation was added in the post-translational modifications along with defaults methionine (M) oxidation and protein N-terminal acetylation; (c) protein intensities were normalized using a minimum ratio count of 1, no Fast LFQ, and no requirement for MS/MS for LFQ comparisons; (d) LC–MS/MS runs were matched under the default matching parameters. All searches were performed against a FASTA file of the *Rattus norvegicus* (canonical and isoforms protein sequences, downloaded from Uniprot on 29-03-2016). Maxquant was configured to automatically average duplicate runs. Peptide and protein identifications were filtered at a 1% false discover rate (FDR) threshold.

### RNA extraction, cDNA synthesis and quantitative RT-PCR (RT-qPCR) analysis

Gene expression analyses were conducted as previously described (Svingen et al. 2015). In short, total RNA was extracted from 9 ovaries/exposure group using the AllPrep kit (#80004; QIAGEN, Manchester, UK) according to the manufacturer’s instructions. RNA concentrations were measured on a nanoDrop-1000 Spectrophotometer and 500 ng RNA/sample used for cDNA synthesis (Omniscript, Qiagen). RT-qPCR reaction were run in duplicates on a QuantStudio 7 Flex Real-Time PCR System (Applied biosystems) in 20 µl reactions using 3 µl diluted cDNA (1:20) as template. TaqMan gene expression assays (Life Technologies) were: *Calb2* (Rn00588816_m1), *Ooep* (Rn01746481_g1), *Hist1h2ba* (Rn00575310_s1), *Snx1* (Rn01418446_m1), *Hdhd2* (Rn01526982_m1), *Eif3a* (Rn01410678_m1), *Hip1* (Rn01202990_m1), *Ufd1l* (Rn00584715_m1), *Lsm14b* (Rn01412698_m1). Intra-assay variability was < 0.5 cycles. Data was analyzed by the comparative Ct-method with the geometric mean of the reference genes *Rps18* (Rn01428913_gH) and *Sdha* (Rn00590475_m1).

### Statistical analysis

MaxLFQ-normalized protein intensities were handled and analyzed in R statistical software (v3.31). Four pairwise statistical comparisons (control vs each of the four exposures) were performed for the proteins with normalized quantitative information in at least 75% of the samples in each comparison using the *limma* package in R. Statistical significance was defined as unadjusted *P* values < 0.05 that showed at least 1.5-fold difference. R code for the analyses performed is available in Online Resource 1. All identified proteins and peptides, including fold differences, unadjusted and adjusted *P* values for all four exposures are provided in Online Resource 2. qPCR data was analyzed by ANOVA and Dunnett’s post-hoc test in the software GraphPad Prism 8. Where data was not normally distributed among all groups, a log transformation was applied.

### Pathway mapping

Ingenuity Pathway Analysis (IPA) V9.0 (Ingenuity Systems, https://www.ingenuity.com) was used to assign affected proteins Disease and Functions pathways (Bellingham et al. 2013; Filis et al. 2015) and to map likely upstream regulators using the differentially expressed proteins as inputs. Predicted disease and functions pathways are shown in Table 2 and upstream regulators in Table 3.

**Table 2:** Disease and function predicted by ingenuity pathway analysis

Categories	Annotation	*P* value	*Z* score	Exposure
Cell death and survival	DNA fragmentation	0.016	− 0.85	Totalmix
Cellular assembly and organization	Formation of cytoskeleton	0.014	− 0.82	Totalmix
Cellular assembly and organization	Formation of filaments	0.018	− 0.82	Totalmix
Cellular assembly and organization	Stabilization of microtubules	0.002	− 0.76	Totalmix
Molecular transport	Transport of molecule	0.001	− 0.59	Totalmix
Cellular assembly and organization	Development of cytoplasm	0.015	− 0.28	Totalmix
Protein synthesis	Catabolism of protein	0.017	0.45	Totalmix
Protein synthesis	Metabolism of protein	0.015	0.82	Totalmix
Cellular movement	Cell movement of mononuclear leukocytes	0.038	1.05	Totalmix
Carbohydrate metabolism	Glycolysis	0.001	1.07	Totalmix
Lipid metabolism	Concentration of lipid	0.027	1.12	Totalmix
Gene expression	RNA transactivation	0.003	1.17	Totalmix
Cell-to-cell signaling and interaction	Adhesion of immune cells	0.025	1.43	Totalmix
Cellular function and maintenance	Internalization of cells	0.014	1.43	Totalmix
Cellular movement	Chemotaxis of leukocytes	0.029	1.67	Totalmix
Cell-to-cell signaling and interaction	Binding of blood cells	0.021	1.70	Totalmix
Cell morphology	Polarization of cells	0.005	1.95	Totalmix
Infectious diseases	Replication of RNA virus	0.006	1.96	Totalmix
Cellular movement	Migration of myeloid cells	0.005	1.98	Totalmix
Cellular movement	Migration of granulocytes	0.010	1.98	Totalmix
Cell morphology	Orientation of cells	0.001	2.18	Totalmix
Cancer	Metastasis of cells	0.025	2.20	Totalmix
Infectious diseases	Replication of Influenza A virus	0.030	2.21	Totalmix
Cell death and survival	Apoptosis	0.041	0.40	Emix
Cell death and survival	Necrosis	0.012	1.09	Emix
Cellular assembly and organization	Microtubule dynamics	0.029	1.41	Emix
Cell-to-cell signaling and interaction	Activation of cells	0.004	− 1.98	PM

**Table 3:** Mapping of upstream regulators by ingenuity pathway analysis

Upstream regulator	Type	*P* value	*Z* score	Exposure
l-Triiodothyronine	Endogenous hormone	0.036	− 1.206	Totalmix
Phosphatase and tensin homolog (Pten)	Phosphatase	0.029	− 1.067	Totalmix
Homebox protein Hox-A10 (Hoxa10)	Transcription regulator	0.013	− 1.000	Totalmix
Progesterone	Endogenous hormone	0.031	− 0.816	Totalmix
Estrogen receptor	Nuclear receptor	0.033	1.000	Totalmix
Matrix metalloproteinase-3 (Mmp3)	Proteinase	0.002	1.000	Totalmix
Vitamin D3	Endogenous chemical	0.002	1.342	Totalmix
Retinoic acid	Endogenous chemical	0.517	1.948	Totalmix
Genistein	Phytoestrogen	0.051	2.177	Totalmix
Interleukin 13 (IL13)	Cytokine	0.044	2.207	Totalmix
Estradiol	Endogenous hormone	0.001	− 0.152	PM

## Results

### Chemical mixture-specific alteration of the ovarian proteome

**Fig. 1:**
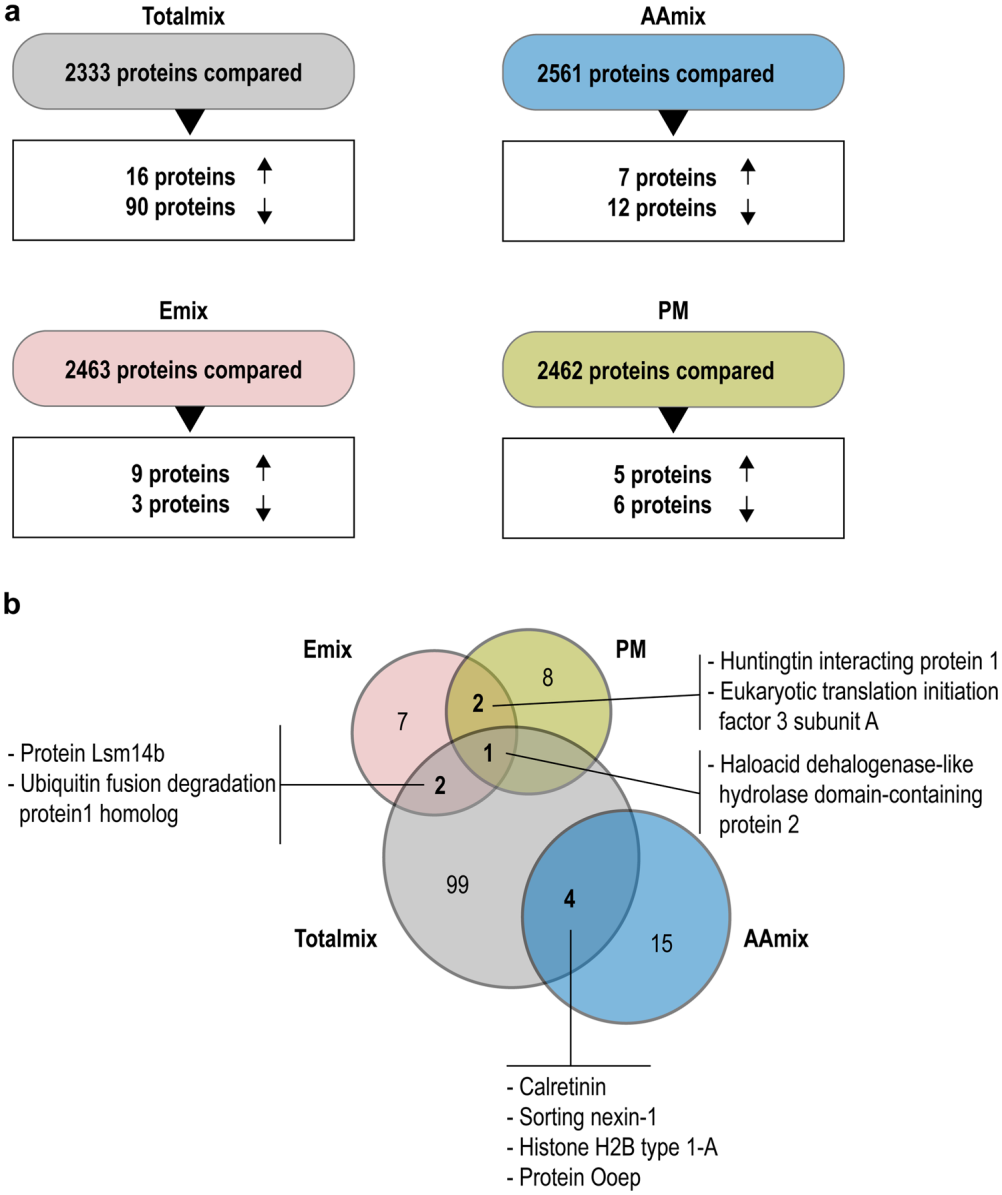
Summary of proteomic findings: **a** Proteome differences between control and Totalmix, AAmix, Emix and PM. For Totalmix 2333 proteins were compared and 16 proteins were upregulated and 90 proteins downregulated. For AAmix 2561 proteins were compared and 7 proteins were upregulated and 12 proteins downregulated. For Emix, 2463 proteins were compared and 9 proteins were upregulated and 3 proteins downregulated. For PM 2462 proteins were compared and 5 proteins were upregulated and 6 proteins down regulated. **b** Venn diagram showing affected proteins overlapping between exposure groups. Totalmix had two upregulated proteins in common with Emix [Protein Lsm14b (LSM14B), Ubiquitin fusion degradation protein 1 homolog (UFD1L)], four proteins in common with AAmix where two were downregulated [Calretinin (CALB2), Sorting nexin-1 (SNX1)] and two upregulated [Histone H2B type 1-A (HIST1H2BA), Protein Ooep (OOEP)]. Totalmix also had one protein in common with both Emix and PM [haloacid dehalogenase-like hydrolase domain-containing protein 2 (HDHD2)]. Emix and PM had two upregulated proteins in common [Huntingtin interacting protein 1 (HIP1), Eukaryotic translation initiation factor 3 subunit A (EIF3A)]. AAmix and PM had no protein overlap (Totalmix: DBP, DEHP, vinclozolin, prochloraz, procymidone, linuron, epoxiconazole, p,pʹ-DDE, 4-MBC, OMC, BPA, Butyl paraben, paracetamol; AAmix: DBP, DEHP, vinclozolin, prochloraz, procymidone, linuron, epoxiconazole, p,pʹ-DDE; Emix: OMC, BPA, Butyl paraben; PM: paracetamol)

After quality filtering there were 2561 distinct proteins in the AAmix group, 2463 in the Emix group, 2462 in the PM group, and 2333 in the Totalmix group. The Totalmix resulted in the highest number of putatively dysregulated proteins in the ovaries (106 proteins), whereas AAmix resulted in 19 proteins with altered expression, the Emix in 12 altered proteins, and Paracetamol 11 altered proteins (Fig. 1a).

Proteins that were changed in Totalmix-exposed ovaries included transcription factors (MED25, NFIC, FOXP4, LOM4, STAT6, and NFIX), proteins involved in the post-transcriptional processing of RNA (TFIP11, DHX57, SKIV2L, THOC6, CMTRL, PCIFL, AQR, ENY2, and DDX39A), cytoskeletal-organization (LCP1, CORO1A, NUDCD3, MAP1B, and STMN1), and proteins associated with intracellular trafficking (GGA1, KIF13A, RAB3GAP2, SNX29, MAPKAPK2, SEC24C, and SNX1). There was also increased expression of CES1, a detoxifying enzyme, and HSD17B12, a key enzyme in ovarian estradiol production. These, and the remainder of proteins displaying altered relative expression, are listed in Online Resource 2.

Proteins that were changed in the AAmix-exposed ovaries included the nuclear transport proteins NUP88 and KPNA1, cytoskeletal organization proteins (SYNPO2, S100A11, MSN and KRT31), and protein involved in metabolism (EIF1B, RBX1, and NPEPL1) (Online Resource 2). In Emix-exposed ovaries, the expression of proteins involved in translation (LSM14B, EIF3A) and signal transduction (ANPEP, LANCL1 and INPPL1) were changed, whereas the proteome of PM-exposed ovaries displayed upregulation of the immunoglobulin lambda chain (LAC) and the pro-apoptotic protein CASP3, as well as changes to expression of the endocytosis factors HIP1 and ANKFY1 (Online Resource 2).

### Common proteins in chemical mixture induced alteration of the ovarian proteome

**Fig. 2:**
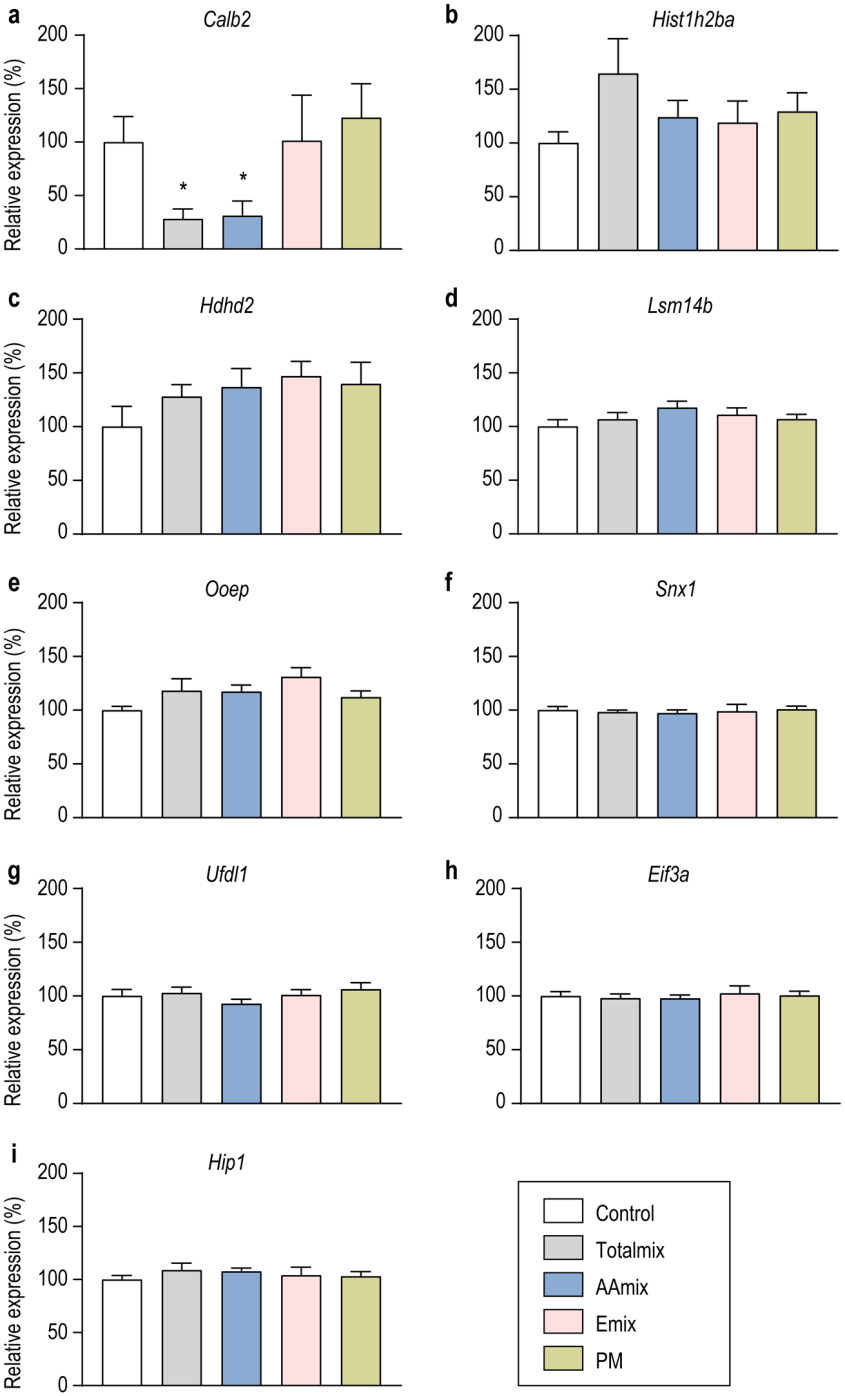
Gene expression analysis for the nine proteins that were dysregulated in more than one exposure group. **a***Calb2* transcript expression was significantly downregulated following exposure to Totalmix (*P* = 0.03) or AAmix (*P* = 0.003). **b–i** No significant effects on expression levels of the remaining 8 genes encoding proteins that were dysregulated in more than one exposure group were seen (data is presented as mean ± SEM, *P < 0.05, **P < 0.01, *n* = 9/exposure group)

Proteins that were altered in more than one exposure group were affected in the same direction; i.e. upregulated or downregulated (Fig. 1b, Online Resource 2). CALB2, SNX1, HIST1H2BA and OOEP were similarly affected in both AAmix and Totalmix with CALB2 expression being reduced 12-fold by Totalmix and fourfold by AAmix (Fig. 1b, Online Resource 2). LSM14B and UFD1L were upregulated after exposure to both Emix and Totalmix whereas HDHD2 was upregulated by exposure to Emix, PM or Totalmix (Fig. 1b, Online Resource 2). AAmix exposed ovaries only showed proteins with altered expression levels in common with Totalmix and not with the other two exposure groups (Fig. 1b). For the nine proteins that were changed in more than one exposure group (Fig. 1b) we chose to investigate gene expression levels. For *Calb2*, a downregulation similar to the one seen at proteome level (12.5 fold downregulation in Totalmix and 4.3 fold downregulation in AAmix) was also found at gene expression level (*P* = 0.03 in Totalmix, *P* = 0.003 in AAmix, Fig. 2a). For the remaining eight proteins, a fold change between 1.6 and 2.4 was seen in the proteome analysis. These changes were not mirrored at the transcript level (Fig. 2b–i).

### Pathway analyses of the affected proteins

Changes to the proteome caused by Totalmix were predicative of increased cancer risk, cell orientation and leucocyte migratory functions in the ovary (Table 2). Totalmix was also predicted to activate pathways relating to infection (replication of RNA viruses), RNA transactivation, and to increase lipid concentration. Changes to the proteome in the Emix group mapped to increase in microtubule dynamics and increased necrosis whereas the paracetamol treatment changes predicted decreased cellular activation (Table 2). Mapping of upstream regulators likely to explain the observed changes to the proteome suggested increased IL13, retinoic acid, Vitamin D3, Matrix Metalloproteinase 3 (Mmp3) and estrogen receptor signaling, whereas it reduced l-triiodothyronine (T3) (Table 3). Downregulated activity for the transcription factor Hoxa10 and of the phosphatase Pten was also predicted after exposure to Totalmix (Table 3).

## Discussion

Exposure to endocrine disrupting chemicals are likely a contributing factor to ovarian dysgenesis syndrome since many chemicals can interfere with both germ cell development and sex hormone synthesis and action (Buck Louis et al. 2011; Johansson et al. 2017). However, because of the multifaceted disease pattern and the fact that humans are exposed to complex mixtures of chemicals, it is challenging to establish cause-effect relationships and to pinpoint the specific molecular events occurring in the young individual that is responsible for the effects seen in the adult. Here we report new knowledge of ovarian molecular pathways, at the level of the proteome, that may be disrupted and responsible for female reproductive disorder and which will contribute to much-needed AOP development.

To discover mechanisms that would cover a broad range of female reproductive disease manifestations arising from exposure to real-life chemical mixtures, we performed shotgun proteomic analyses on rat ovaries obtained from juvenile offspring exposed to four different chemical mixtures comprising known EDCs during fetal and postnatal life. By doing so, we could investigate effects on thousands of proteins after exposure to complex chemical mixtures more resembling human exposure patterns than what is the case for single chemical exposures. We found several differentially expressed proteins across the four exposure groups, with nine proteins being altered in more than one exposure group (CALB2, SNX1, HIST1H2BA, OOEP, LSM14B, UFD1L, HDHD2, HIP1, EIF3A). This is promising in view of detecting a molecular initiating event or key event that can potentially serve as a reference point for a wide range of chemicals. As expected, some of the proteins altered in the Totalmix group were also altered in other exposure groups. Also, the Emix and PM groups showed proteins with altered expression in common with each other, whereas AAmix only had misexpressed proteins in common with Totalmix, separating this group from Emix and PM. This is of particular interest as Totalmix and AAmix showed marked long-term effects on the ovary in a previous study (Johansson et al. 2016). Of the various proteins, calretinin (CALB2) displayed greatest change in relative expression level, and the effect was replicated at the transcript level.

Calretinin is a calcium binding protein that buffers the Ca^2+^ levels in the cell (Schwaller 2014). Calretinin was first identified in the nervous system (Rogers 1987; Andressen et al. 1993) and most research into its function has been carried out on brain tissues (Schwaller 2014). However, calretinin is also expressed in non-excitable cells in several other tissues, including the ovary where it is expressed in the theca interna of follicles and theca lutein cells in corpora lutea (Bertschy et al. 1998; Lugli et al. 2003). In relation to the female reproductive system, calretinin is best known as a diagnostic marker for several types of gynecological tumors, where it is ectopically activated (Portugal and Oliva 2009). In the present study, calretinin was downregulated in ovaries after exposure to mixtures containing anti-androgenic chemicals (Totalmix and AAmix). Since the theca cells are the androgen producing cells of the ovary, and the cells that express calretinin (Bertschy et al. 1998; Lugli et al. 2003), there may be two explanations for the downregulation of calretinin following chemical exposure. One, theca cell differentiation or maintenance may be affected so that their overall numbers are reduced; in other words, intracellular calretinin is not affected but there are fewer cells in the ovary expressing calretinin. Two, calretinin expression is directly affected, indicating compromised theca cell function. In the fetal testis, calretinin levels are correlated with the number of Leydig cells (Altobelli et al. 2017). Since ovarian theca cells corresponds to testicular Leydig cells, at least in terms of androgen production, similar functions for calretinin in the ovary are likely, which would lend support to the first scenario where it is the theca cell numbers that are affected.

Also, recent findings suggest that calretinin is involved in Leydig cell function by protecting against cell apoptosis (Xu et al. 2017) and by promoting steroidogenesis (Xu et al. 2018). Thus, the potential importance of calretinin in androgen producing cells, as well as the effects on primordial follicles and reproductive senescence seen in littermates exposed to anti-androgenic mixtures (Johansson et al. 2016), indicates that calretinin could be a potential marker not only of anti-androgenic effect, but potentially also for late life ovarian dysgenesis.

Mapping of upstream regulators showed interesting results with regulation through molecules such as retinoic acid, phosphatase and tensin homolog, and vitamin D3. Whether or not any of these proteins are actually molecular initiating events or key events remains unanswered, but they could serve as starting points for further investigations. As an example, retinoic acid plays a central role in germ cell development and particularly meiotic initiation (Koubova et al. 2006; Bowles et al. 2006).

The ovary is a dynamic organ undergoing dramatic changes during the menstrual/estrous cycle in adult individuals and also during pre-pubertal development (Picut et al. 2015). This, coupled with the fact that many changes to the ovaries caused by chemical exposure likely would have occurred early in life (Johansson et al. 2017), makes it difficult to catch causative changes to protein expression. Thus, timing is a critical factor in the search for molecular initiating, or key events, especially when also considering that the ovary is a heterogeneous tissue. Omics approaches are good avenues for searching for such mechanisms, but they also have their drawbacks. With regard to shotgun proteomics, it must be noted that only the most abundant proteins are detected (Cayer et al. 2016), and not necessarily those with the biggest changes in expression levels or that are functionally relevant for the diseases in question. In this study, we have allowed for less stringent adjusted p-value cut offs, a practice not uncommon when dealing with in vivo proteomic data sets (Pascovici et al. 2016), and to reflect biological relevance and to mitigate Type 1 statistical errors we have used fold difference cut offs. Our reported list of affected proteins may therefore contain false-positives, but at the same time it likely contains important protein markers that describe mechanisms of ovarian dysgenesis. The nine proteins we chose for further investigation may be an excellent example of this. We found effects on transcriptional regulation of *Calb2* (calretinin), whereas we did not see any effects on *Ooep*, *Hist1h2ba*, *Snx1*, *Hdhd2*, *Eif3a*, *Hip1*, *Ufd1l*, *Lsm14b*. This could indicate that these eight proteins are regulated at post-transcriptional level, or that they represent false positives. Regardless, rigorous follow-up studies on the reported protein targets is required to pass judgment on their bona fide involvement in disease causation or development.

Overall, our study has highlighted calretinin as a potentially interesting target for many of the female disorders that fall under the ovarian dysgenesis syndrome umbrella. Since we observed a significant downregulation of calretinin both at the protein and transcript level, we conclude that calretinin is a strong candidate for a key event in ovarian dysgenesis syndrome. Downregulation of calretinin was seen in the Totalmix and AAmix groups, indicating that altered expression of calretinin is a direct consequence of anti-androgenic effects. This data warrants further studies of calretinin’s role in signaling pathways in the developing as well as the adult ovary, directed towards much-need ovarian AOP development.

## Electronic supplementary material

Below is the link to the electronic supplementary material.

Supplementary file1 (PDF 264 kb)

Supplementary file2 (XLSX 843 kb)
