# Supplementary material for: Calretinin is a novel candidate marker for adverse ovarian effects of early life exposure to mixtures of endocrine disruptors in the rat
Source: Arch Toxicol. 2020 Mar 27;94(4):1241–50. doi: 10.1007/s00204-020-02697-3 (PMC7225203; doi:10.1007/s00204-020-02697-3)
Supplement: Supplementary file 1 — Supplementary file1 (PDF 264 kb) [file 204_2020_2697_MOESM1_ESM.pdf]

“Calretinin is a novel candidate marker for adverse ovarian effects of early life exposure to mixtures of endocrine disruptors in the rat”

Archives of Toxicology

Hanna Katarina Lilith Johansson, Terje Svingen, Julie Boberg, Paul A. Fowler, David Stead, Anne Marie Vinggaard, Panagiotis Filis

(Corresponding author Panagiotis Filis, Institute of Medical Sciences, School of Medicine, Medical Sciences & Nutrition, University of Aberdeen, Foresterhill, Aberdeen, AB25 2ZD, UK, Email: pfilis@abdn.ac.uk)

Online Resource 1 Example of R-code used for statistical analysis of the MaxLFQ-normalized protein intensities in R statistical software (v3.31). Four statistical comparisons (control vs each of the four treatments) were performed for the proteins with normalized quantitative information in at least 75% of the samples in each comparison using the *limma* package in R

```
library(limma)

#import protein dataframe
prots.raw <- read.delim("Maxquant output.txt", row.names=1, header=TRUE) ##
dim(prots.raw)

#convert 0 values to NA
prots.raw[prots.raw ==0]<-NA


###remove columns with more than 25% NA values
prots.raw1<-prots.raw[, colMeans(is.na(prots.raw)) <= 0.25]
dim(prots.raw1)

#drop 1st column (treatment info) & log2 convert protein intensities
prots.log<-log2(prots.raw1[,-1])


############        LIMMA        #######

###create phenotypic (treatment) dataframe
pheno <- data.frame(cbind(TREATMENT = c(rep("C",8), rep("X.treatment",8))), row.names=rownames(prots.log))

mod <- model.matrix(~TREATMENT, data=pheno)
fit<- lmFit(t(prots.log), mod)
fit2<-eBayes(fit)
results<- decideTests(fit2, adjust.method="none", method="global", p=0.05)
summary(results)

topTable(fit2, coef=c(2),n =10, adjust.method="BH")

# extract 2nd coefficient (treatment)
res<-topTable(fit2, coef=c(2),n =Inf, adjust.method="BH")

write.table(res, "C vs X.treatment.csv", sep=",")
```
